# Population genetic structure of the messmate pipefish *Corythoichthys haematopterus* in the northwest pacific: evidence for a cryptic species

**DOI:** 10.1186/2193-1801-2-408

**Published:** 2013-08-28

**Authors:** Atsushi Sogabe, Motohiro Takagi

**Affiliations:** Graduate School of Biosphere Science, Hiroshima University, 1-4-4 Kagamiyama, Higashi-Hiroshima, 739-8528 Japan; South Ehime Fisheries Research Center, Tarumi Branch, Ehime University, 3-5-7 Tarumi, Matsuyama, 790-8566 Japan; Center for Marine Biology, Asamushi, Tohoku University, 9 Sakamoto Asamushi, Aomori, 039-3501 Japan

**Keywords:** Cryptic species, Molecular phylogeny, Syngnathidae, Phylogeography, *Corythoichthys haematopterus*

## Abstract

The population genetic structure of the messmate pipefish, *Corythoichthys haematopterus*, in the northwest Pacific was investigated based on the partial mitochondrial DNA cytochrome *b* (589 bp) and 16S rRNA (528 bp) region sequences of 108 individuals collected from six sites along the coast of the Japanese archipelago and one site on Mactan Island, the Philippines. A total of 60 and 28 haplotypes were obtained from the cytochrome *b* and 16S rRNA regions, respectively. Two genetically distinct lineages were detected: lineage A and B, which are separated by mean pairwise genetic distances of 23.3 and 14.1% in the partial cytochrome *b* and 16S rRNA genes, respectively. Such a huge genetic divergence between lineages, which is comparable to or even higher than the interspecific level, and the difference in their geographical distributions and habitat preferences suggests that they are distinct species, although there is no marked difference in their morphology. Haplotype network and gene and nucleotide diversity statistics indicate that the two lineages have different biogeographic histories: lineage A experienced rapid population expansion after a population bottleneck whereas lineage B has a long evolutionary history in a large stable population. In contrast, the levels of genetic variation among populations are relatively low in both lineages, probably because of frequent gene flow among populations resulting from the dispersal of pelagic larvae by the Kuroshio Current. These results indicate that past climatic events and contemporary oceanographic features have played a major role in establishing the population genetic structure of *C. haematopterus*.

**Electronic supplementary material:**

The online version of this article (doi:10.1186/2193-1801-2-408) contains supplementary material, which is available to authorized users.

## Introduction

Elucidating the population genetic structure of a species can aid conservation decisions, enable us to identify demographically independent populations that should be managed as separate units (i.e. Evolutionarily Significant Unit, ESU; Fraser and Bernatchez 2001), and assess connectivity among local populations thus providing useful information for reservation design (Palumbi 2003). In contrast to terrestrial organisms, the population structure of marine organisms has long been assumed to be homogenous, because most of them have long pelagic larval durations and there are no obvious geographical barriers in marine environments. However, increasing evidence shows that significant genetic structure does exist in marine species, implying that both past geological and climatic events (e.g. plate tectonic movement or glacial episodes) and present oceanographic features (e.g. ocean currents) have undoubtedly played a major role in marine biogeography (Jones et al. 1999; Barber et al. 2000; Lourie and Vincent 2004; Wilson 2006; von der Heyden et al. 2011).

High population genetic structure is expected to develop when gene flow among populations is severely restricted. This is the case for marine organisms, especially sessile or sedentary species, when larval dispersal is prevented by short pelagic larval duration or oceanographic features, or when migrating larvae have no chance of growing and entering non-natal breeding grounds because of crucial differences in selection regimes among populations (i.e. local adaptation), even though transportation of larvae may occur (Palumbi 2003). In the northwest Pacific, two strong ocean currents, the Kuroshio and North Equatorial Currents, should facilitate the dispersal of pelagic larvae. The Kuroshio Current originates in the westward-flowing North Equatorial Current of the central Pacific Ocean and deflects towards the northeast offshore of the Philippine Islands. The main axis of the Kuroshio Current enters the Okinawa Trough northeast of Taiwan Island with a maximum speed of 100 cm/s and a width of 100 km, and flows northeastward along the Japanese archipelago (Liang et al. 2003; Andres et al. 2008). Thus, it would be expected that ample gene flow from southern to northern populations reduces the spatial component of genetic variability among populations. Nonetheless, there are many examples that marine organisms having a pelagic larval phase show strong regional genetic differentiation in the area of the Kuroshio Current (Ogoh and Ohmiya 2005; Kojima et al. 2006; Liu et al. 2008; Yorifuji et al. 2012).

Syngnathid fishes (pipefishes, seahorses, and seadragons), in which males provide all parental care to the developing eggs that are glued to the male’s body or brooded in specialized pouch (Wilson et al. 2001), are thought to have limited dispersal ability because the swimming capacity of adults is low and some lack a pelagic larval dispersal phase (Lourie and Vincent 2004). Consequently, they can show a high degree of genetic divergence among populations and adaptation to local environments (Lourie et al. 2005). At present, many syngnathid species are faced with overexploitation for the aquarium and traditional medicine trades, habitat degradation or loss as a result of anthropogenic activity (Vincent 1996). Therefore, gaining a better understanding of their biology, including population genetics, is a pressing issue.

The messmate pipefish *Corythoichthys haematopterus* is widely distributed in the shallow waters of the Indo-Pacific (Dawson 1977; Nakabo 2002). For syngnathid fish, *C. haematopterus* is thought to have a long pelagic duration (ca. 1 month), judging from the timing of juvenile recruitment and the morphology of hatchlings. Adult fish show strong site fidelity and stay within a small home range (often less than 100 m^2^) for their entire lives (Matsumoto and Yanagisawa 2001). This indicates that dispersal only occurs during the pelagic larval phase and is thus expected to be highly dependent on ocean currents. The aim of this study is to determine the contemporary population genetic structure of *C. haematopterus* in the northwest Pacific using mitochondrial DNA markers (cytochrome *b* and 16S rRNA) to examine the potential influence of the Kuroshio Current on dispersal and genetic connectivity among *C. haematopterus* populations. A preliminary study revealed that there is geographical variation in *C. haematopterus* habitat preferences; the habitat of the main Japan islands population is a steep slope consisting of boulder and bedrock at around 2–10 m depth, whereas that of the Ryukyu Islands population is a sandy bottom partly covered with seagrass meadows in a shallow (less than 1 m depth) reef lagoon (A. Sogabe unpubl data). We therefore examined if such geographical variation equates to genetic differentiation as a consequence of local adaptation.

## Methods and materials

### Sample collection

**Figure 1:**
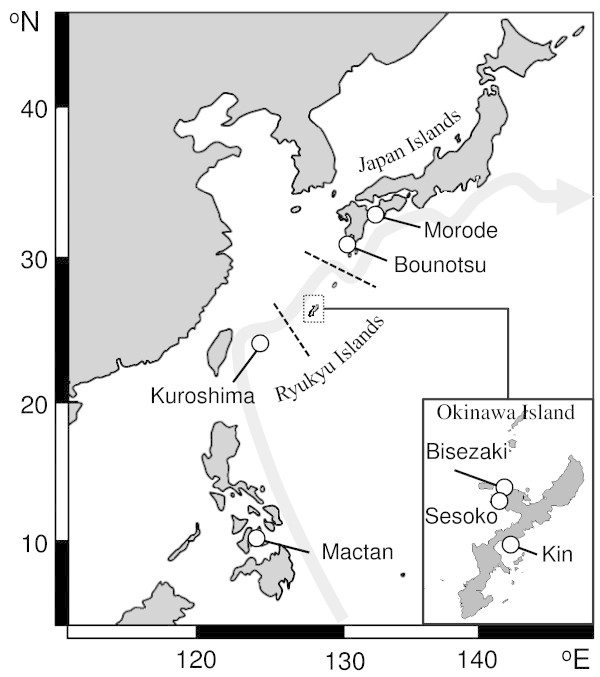
**Map of sampling sites of*Corythoichthys haematopterus.****Corythoichthys haematopterus* sampling sites. The arrow indicates the path of the Kuroshio Current and the dash lines indicate the Tokara (north) and Kerama Gaps (south).

A total of 108 *Corythoichthys haematopterus* individuals were collected with a hand net from six sites along the coast of the Japanese archipelago and one site on Mactan Island, the Philippines (Figure 1). There was great variation in habitat preference among the populations: *C. haematopterus* inhabited steep slopes (2–10 m depth) consisting of boulder and bedrock in Morode and Bounotsu, whereas they resided in shallow reef lagoons (less than 1 m depth) partly covered with seagrasses (e.g., *Thalassia hemprichii*) in Bisezaki, Sesoko, Kuroshima, and Mactan. In Kin, however, they occurred on the vertical surface of a wharf constructed on a sandy beach (0–7 m depth). Although four species of *Corythoichthys* pipefish (*C. amplexus*, *C. flavofasciatus*, *C. haematopterus*, and *C. schultzi*) are known to inhabit the coast of the Japanese archipelago, they are easily distinguished from each other based on their coloration and snout to postorbital length ratio (Nakabo 2002). A picture of the whole body of each fish was taken to measure the standard length and a clipping from the caudal fin were taken in the field, they were then released back to the sample site. Some fish were euthanized with clove oil (Wako Pure Chemical Industries Ltd., Osaka, Japan) for detailed morphological assessment (see below). The caudal fins were preserved in 99.5% ethanol. We also collected three *C. amplexus*, three *C. schultzi*, and one *Doryrhamphus dactyliophorus* individual at Sesoko for phylogenetic analyses. This work adhered to the Animal Behaviour Society guidelines for the use of animals in research (Animal Behaviour 1998).

### DNA extraction, PCR, and sequencing

Total DNA was extracted from the fin tissue preserved in 99.5% ethanol using the QuickGene DNA Tissue Kit S (Fujifilm Corporation, Tokyo, Japan), according to the manufacturer’s instructions, and stored at −4°C. A partial sequence of the mitochondrial cytochrome *b* gene and the mitochondrial 16S ribosomal RNA (16S rRNA) was amplified using the primers L14725 and H15926, and L2510 and H3058, respectively (Table 1). The fragments were amplified by polymerase chain reaction (PCR) under the following conditions; an initial denaturation for 5 min at 96°C, followed by 40 cycles of 94°C (1 min), 48°C (1 min), 72°C (1 min), and a final extension at 72°C for 4 min for cytochrome *b*. The conditions for 16S rRNA were as follows; an initial denaturation for 6 min at 94°C, followed by 30 cycles of 94°C (45 s), 55°C (45 s), 72°C (2 min), and a final extension at 72°C for 6 min. The PCR products were purified with ExoSAP-IT (GE Healthcare U.K. Ltd., Little Chalfont, UK). DNA sequencing was performed using the BigDye® terminator v3.1 Cycle Sequencing Kit (Applied Biosystems, Warrington, UK) and an ABI 3100 automated DNA sequencer (Applied Biosystems). The primer used for sequencing the partial 16S RNA was the same as that used for the PCR. However, we designed two internal primers, Coh-F43 and Coh-L80 for sequencing the partial cytochrome *b* gene (Table 1). Sequences were proofread and first aligned with ClustalW (Chenna et al. 2003); the alignments were then verified by eye and trimmed. All unique haplotypes were deposited in DDBJ (accession numbers: 16S ribosomal RNA, AB827829–AB827865; cytochrome *b*, AB827866–AB827925).

**Table 1:** **PCR primers used in the study**

Primer	Sequence	Reference
Cytochrome *b*		
L14725	5’–CGAAGCTTGATATGAAAAACCATCGTTG–3’	Pääbo et al. 1991
Coh-F43	5’–GCCTTCGAACATCTCAGTCTG–3’	This study
Coh-L80	5’–GAAACTTCGGCTCACTACTCG–3’	This study
H15926	5’–AAGGGKGGATTTTAACCTCCG–3’	Wilson et al. 2001
16S		
L2510	5’–CGCCTGTTTATCAAAAACAT–3’	Palumbi et al. 1991
H3058	5’–CCGGTCTGAACTCAGATCACGT–3’	Palumbi et al. 1991

### Data analysis

Data from Bisezaki and Sesoko were pooled for analyses, because these sites were only 8 km apart. Standard gene diversity (*h*) and nucleotide diversity (***π***) statistics were calculated for each sampling site and for each locus using Arlequin 3.5 (Excoffier and lischer 2010). Pairwise *F*_ST_s were computed to examine the genetic heterogeneity among populations; significance was tested with 10, 000 permutations of the data set in Arlequin. A correlation between genetic (as *F*_ST_) and geographic distance was examined by a Mantel test (Mantel 1967; Smouse et al. 1986), and the statistical significance was calculated by permuting the data for 10, 000 replicates using Arlequin. Geographic distance between sites was calculated using the shortest straight-line sea distance. Parsimony-based haplotype networks were constructed for both loci with the program Tcs 2.1 (Clement et al. 2000).

Phylogenetic trees were constructed by the neighbor-joining (NJ) method (Saitou and Nei 1987), using Mega5 (Tamura et al. 2011). Two *Syngnathus* pipefish, *S. typhle* (GenBank accession number: AF356042) and *S. scovelli* (AF356068), were used as outgroups for partial cytochrome *b-*based phylogenetic analysis, and *Doryrhamphus dactyliophorus* collected in the present study as an outgroup for partial 16S rRNA-based phylogenetic analysis. Cytochrome *b* and 16S rRNA sequences of other congeneric pipefishes extracted from the GenBank database (cytochrome *b*: *C. intestinalis* sampled in Guam, accession number AF356055; *C. intestinalis* sample in Indonesia, AF356052; *C. haematopterus* sampled in Japan, AY166830; 16S rRNA: *C. intestinalis* sampled in Guam, AF355005; *C. intestinalis* sample in Indonesia, AF355003; *C. haematopterus* sampled in Japan, AY166831), obtained from a museum (National Museum of Nature and Science, Japan; *C. flavofasciatus*: specimen voucher: NSMT-P 110434 and BSKU-108281), and collected in the present study (*C. amplexus*, *C. schultzi*) were also included in the analyses. *Corythoichthys amplexus*, *C. flavofasciatus*, and *C. schultzi* were only included in the phylogenetic analysis based on 16S rRNA sequences because of the difficulty in sequencing the cytochrome *b* region. Preliminary sequence analyses using Mega indicated that the cytochrome *b* fragment best fits an Tamura-Nei model with gamma shape parameter = 0.24 (TN93 + ***Γ***; Tamura and Nei 1993) and the 16S rRNA fragments best fits an Kimura 2-parameter model with gamma shape parameter = 1.03 and assumption that a certain fraction of sites are evolutionarily invariable (K2P + ***Γ*** Kimura  + *I*; 1980). The evolutionary distances were computed using those models, and topological confidence was evaluated with 1,000 bootstrap replicates (Felsenstein 1985).

### Morphological characterization

Morphological assessment was conducted in three individuals each from Morode, Kin, Bisezaki, and Kuroshima, and all fish collected in Mactan (*n* = 11). Fish were measured for standard length (SL) to the nearest 0.1 mm using a slide caliper. The number of fin rays on the dorsal, pectoral, and caudal fins and the number of body rings (i.e., trunk and tail rings) were counted under a microscope.

## Results

### Cytochrome *b* and 16S rRNA sequence analysis

Partial nucleotide sequences of the cytochrome *b* (589 bp) and 16S rRNA regions (528 bp) were determined for a 108 *Corythoichthys haematopterus* individuals. Alignment of partial cytochrome *b* sequences detected 136 variable sites without any indels, resulting in 60 haplotypes (Additional file 1). In contrast, of the 16S rRNA sequences detected 75 variable sites included 3 indels and resulted in 28 haplotypes (Additional file 2). Gene (*h*) and nucleotide diversity (*π*) ranged from 0.844 to 0.977 and 0.0025 to 0.0930, respectively, for partial the cytochrome *b* region. For the partial 16S rRNA region gene (*h*) and nucleotide diversity (*π*) ranged from 0.538 to 0.895 and 0.0012 to 0.0625, respectively (Table 2). Both gene and nucleotide diversity estimates were highest in Kin in both mitochondrial regions, but the lowest values were found in the Morode and Bounotsu populations for cytochrome *b* and 16S rRNA, respectively.

**Table 2:** **Summary of mitochondrial DNA data for six*Corythoichthys haematopterus*populations**

			Cytochrome ***b***				16S rRNA			
Sampling site	No.	Standard length	No.	No.	Gene diversity	Nucleotide diversity	No	No.	Gene diversity	Nucleotide diversity
	gen.	(mm, mean ± SD)	hap.	uni.	(***h*** ± SD)	(***π*** ± SD)	hap.	uni.	(***h*** ± SD)	(***π*** ± SD)
Morode	22	131.5 ± 10.3	10	8	0.844 ± 0.062	0.0025 ± 0.0018	8	3	0.546 ± 0.128	0.0012 ± 0.0011
(33°00'N, 132°30'E)										
Bounotsu	19	115.1 ± 10.2	11	7	0.889 ± 0.058	0.0029 ± 0.0020	6	1	0.538 ± 0.133	0.0012 ±0 .0011
(31°20'N, 130°12'E)										
Kin	19	114.6 ± 10.1	16	11	0.977 ± 0.027	0.0930 ± 0.0470	10	7	0.895 ± 0.048	0.0625 ± 0.0319
(26°26'N, 127°54'E)										
Bisezaki*	19^*^	128.8 ± 3.3^†^	15	12	0.959 ± 0.036	0.0097 ± 0.0054	6	4	0.678 ± 0.093	0.0020 ± 0.0015
(26°42'N, 127°53'E)										
Kuroshima	18	109.3 ± 14.5	11	7	0.909 ± 0.051	0.0064 ± 0.0038	5	3	0.601 ± 0.113	0.0017 ± 0.0014
(24°15'N, 124°01'E)										
Mactan	11	109.2 ± 6.2	8	7	0.927 ± 0.067	0.0109 ± 0.0063	4	3	0.709 ± 0.099	0.0048 ± 0.0031
(10°16'N, 123°59'E)										
Total	108		60	-	0.960 ± 0.011	0.0898 ± 0.0433	28	-	0.840 ± 0.023	0.0599 ± 0.0292

Sampling sites, number of individuals genotyped (No. gen.), mean standard length (mm ± SD), number of haplotypes (No. hap.), number of unique haplotypes (No. uni.), gene diversity (*h* ± SD), and nucleotide diversity (*π* ± SD) statistics are listed.

*Including four individuals collected in Sesoko; †Based on 3 individuals collected in Bisezaki.

**Figure 2:**
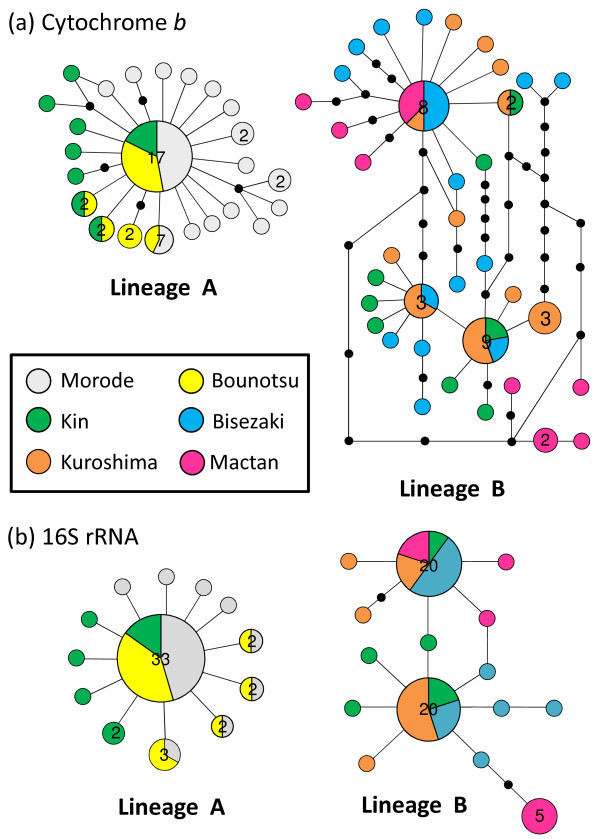
**Statistical parsimony haplotype network for two mitochondrial regions of*Corythoichthys haematopterus.*** Statistical parsimony network for **a)** the partial cytochrome *b* region (589 bp) and **b)** the partial 16S rRNA region (528 bp) haplotypes identified in *Corythoichthys haematopterus*. Each connection is a single mutational step, and small black circles represent inferred haplotypes. The numbers in the circles indicate the number of individuals (≥2 individuals) with that haplotype.

Most of the population pairwise *F*_ST_ tests were significant, with non-significant pairwise tests occurring between Morode and Bounotsu and Bisezaki and Kuroshima for both mitochondrial regions (Table 3). There was a significant correlation between geographic and genetic distance (as pairwise *F*_ST_s) for both the cytochrome *b* and 16S rRNA regions (Mantel test: cytochrome *b*: *r* = 0.35, p = 0.048; 16S rRNA: *r* = 0.35, p = 0.046). A statistical parsimony haplotype network is shown in Figure 2. The different haplotypes did not join into a single network with 95% confidence, and we identified two clearly distinctive lineages, separated by a huge number of mutational steps for both regions (ca. 100 and 60 bp substitution for the partial cytochrome *b* and 16S rRNA regions, respectively; see also Additional files 1 and 2). One lineage comprised haplotypes from Morode, Bounotsu, and Kin (Haplotype 1–24 for cytochrome *b* and 1–13 for 16S rRNA; hereafter lineage A), and the other comprised haplotypes from Kin, Bisezaki, Kuroshima, and Mactan (haplotype 25–60 for cytochrome *b* and 14–28 for 16S rRNA; hereafter lineage B). Within lineage A, the most common haplotype (haplotype 10 for cytochrome *b* and 8 for 16S rRNA) related by one or two mutational steps to numerous singleton haplotypes and five haplotypes of moderate frequency (found in two to seven individuals) for both mitochondrial regions were observed. Within lineage B, however, there were two main haplotypes (haplotype 31 and 45 for cytochrome *b* and 17 and 18 for 16S rRNA), which were connected by either one or several mutational steps to each other.

**Table 3:** **Population pairwise*F***_**st**_**s of six populations of*Corythoichthys haematopterus***

	Morode	Bounotsu	Kin	Bisezaki	Kuroshima	Mactan
Morode		- / -	+ / +	+ / +	+ / +	+ / +
Bounotsu	−0.001 / -0.0297		+ / +	+ / +	+ / +	+ / +
Kin	0.44606 / 0.45254	0.42194 / 0.42976		+ / +	+ / +	+ / +
Bisezaki	0.96612 / 0.98673	0.96322 / 0.98657	0.45889 / 0.48627		- / -	+ / +
Kuroshima	0.97533 / 0.98776	0.97314 / 0.98769	0.46439 / 0.48115	0.06622 / 0.01605		+ / +
Mactan	0.96931 / 0.9798	0.96596 / 0.97886	0.4045 / 0.41182	0.16684 / 0.23522	0.33559 / 0.27719	

Population pairwise *F*_st_ estimates between sampling sites based on the partial cytochrome *b* (*left*) and 16S rRNA (*right*) regions of Cor*ythoichthys haematopterus*. + and – indicate significant and non-significant differences at P < 0.05, respectively.

**Figure 3:**
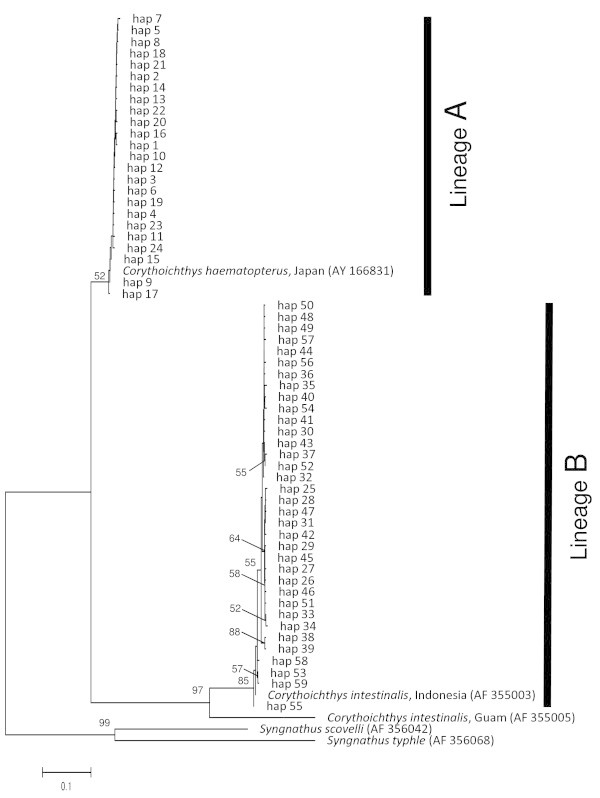
**Neighbor-joining tree inferred by the partial cytochrome*b*gene for*Corythoichthys haematopterus.*** Neighbor-joining tree based on Tamura-Nei methods, inferred by the partial cytochrome *b* gene (589 bp) for haplotypes of the *Corythoichthys haematopterus* lineage **A** and **B** (see text) with *Syngnathus scovelli* and *S. typhle* as outgroups. Numbers associated with the branches indicate bootstrap replicate values (%) for groupings supported by values >50%.

**Figure 4:**
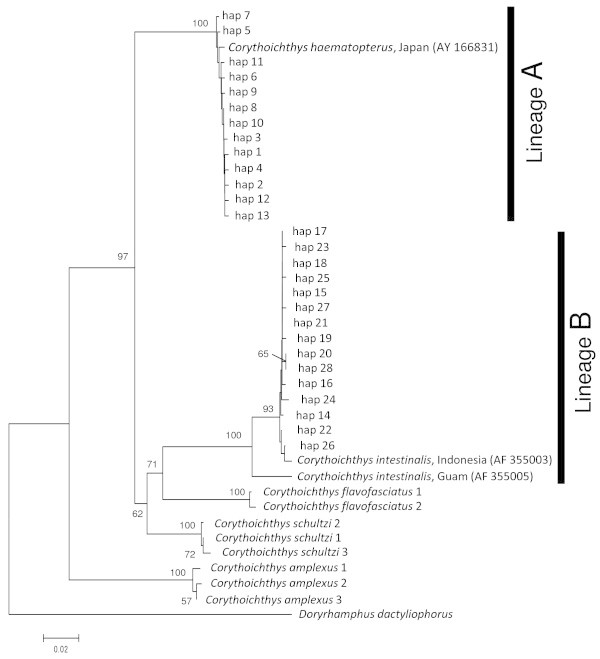
**Neighbor-joining tree inferred by the partial 16S rRNA gene for*Corythoichthys haematopterus.*** Neighbor-joining tree based on Kimura 2-parameter methods, inferred by the partial 16S rRNA gene (472 bp) for haplotypes of the *Corythoichthys haematopterus* lineage **A** and **B** (see text) with *Doryrhamphus dactyliophorus* as outgroup. Numbers associated with the branches indicate bootstrap replicate values (%) for groupings supported by values >50%.

The NJ trees based on genetic distances estimated from the partial cytochrome *b* and 16S rRNA gene sequences also revealed two distinct lineages with high bootstrap probabilities (Figures 3 and 4). Lineage A (haplotype 1–24 for cytochrome *b* and 1–13 for 16S rRNA) comprised 51 individuals collected in Morode, Bounotsu, and Kin, and lineage B (haplotype 25–60 for cytochrome *b* and 14–28 for 16S rRNA) was composed of 57 individuals from Kin, Bisezaki, Kuroshima, and Mactan. Mean pairwise genetic distances between two lineages were 23.3 and 14.1% in the partial cytochrome *b* and 16S rRNA genes, respectively, whereas intra-lineage genetic distance was extremely low for both regions (0.4 and 1.2% in cytochrome *b*, and 0.4 and 0.5% in 16SrRNA). For the partial 16S rRNA gene, mean pairwise genetic distance between lineages was comparable to or higher than that among congeneric species (lineage A: vs. *C. flavofasciatus* 12.6%, vs. *C. schultzi* 9.3%, vs. *C. amplexus* 16.8%; lineage B: vs. *C. flavofasciatus* 12.3%, vs. *C. schultzi* 11.5%, vs. *C. amplexus* 19.7%). The phylogenetic tree based on 16S rRNA revealed that *C. schultzi* and *C. flavofasciatus* are sister groups to lineage B and thus *C. haematopterus* collected in this study was a paraphyletic group. Unexpectedly, the *C. intestinalis* (sampled in Indonesia) sequence data extracted from GenBank was included in a branch of lineage B, although that of *C. haematopterus* (sampled in Japan) was included in a branch of lineage A (Figures 3 and 4).

### Population genetic structure of each lineage

We reanalyzed the genetic divergence and population structure of *Corythoichthys haematopterus* by separating it into two lineages. In lineage A, gene (*h*) and nucleotide diversity (*π*) ranged from 0.844 to 0.933 and 0.0025 to 0.0033, respectively, for the partial cytochrome *b* region, and from 0.538 to 0.756 and 0.0012 to 0.0018, respectively, for the partial 16S rRNA (Table 4). Both gene and nucleotide diversity estimates were highest in the Kin population in both mitochondrial regions, but the lowest values were observed in the Morode population for cytochrome *b* and the Bounotsu population for 16S rRNA (Table 4). Population pairwise *F*_ST_ tests were all non-significant (Table 5). In lineage B, the gene (*h*) and nucleotide (*π*) diversity ranged from 0.909 (Kuroshima) to 0.973 (Kin) and 0.0064 (Kuroshima) to 0.0109 (Mactan), respectively, for the partial cytochrome *b* region, and from 0.601 (Kuroshima) to 0.806 (Kin) and 0.0017 (Kuroshima) to 0.0048 (Mactan), respectively, for the partial 16S rRNA (Table 4). Population pairwise *F*_st_ tests confirmed significant genetic divergence between Mactan and the other sites for both mitochondrial regions (Table 5). In contrast, Mantel tests revealed that there was no significant correlation between genetic and geographic distance for either the cytochrome *b* or the 16S rRNA region in both lineages whereas the correlation coefficients were relatively high (lineage A: cytochrome *b*: *r* = 0.99, p = 0.16; 16S rRNA: *r* = 0.85, p = 0.33; lineage B: cytochrome *b*: *r* = 0.78, p = 0.21; 16S rRNA: *r* = 0.92, p = 0.21).

**Table 4:** **Summary of mitochondrial DNA data for sampled populations of*Corythoichthys haematopterus*lineage A and B (see text and Figures**3**and**4**)**

Lineage	Sampling site		Cytochrome ***b***				16S rRNA			
		No.	No	No.	Gene diversity	Nucleotide diversity	No.	No.	Gene diversity	Nucleotide diversity
		gen.	hap.	uni.	(***h*** ± SD)	(***π*** ± SD)	hap.	uni.	(***h*** ± SD)	(***π*** ± SD)
A	Morode	22	10	8	0.844 ± 0.062	0.0025 ± 0.0018	8	3	0.546 ± 0.128	0.0012 ± 0.0011
	Bounotsu	19	11	7	0.889 ± 0.058	0.0029 ± 0.0020	6	1	0.538 ± 0.133	0.0012 ± 0.0011
	Kin	10	8	5	0.933 ± 0.077	0.0033 ± 0.0023	5	4	0.756 ± 0.130	0.0018 ± 0.0015
	Total	51	24	-	0.873 ± 0.040	0.0028 ± 0.0019	13	-	0.580 ± 0.082	0.0013 ± 0.0011
B	Kin	9	8	6	0.973 ± 0.064	0.0069 ± 0.0043	5	3	0.806 ± 0.120	0.0019 ± 0.0016
	Bisezaki	19	15	12	0.959 ± 0.036	0.0097 ± 0.0054	6	4	0.678 ± 0.093	0.0020 ± 0.0015
	Kuroshima	18	11	7	0.909 ± 0.051	0.0064 ± 0.0038	5	3	0.601 ± 0.113	0.0017 ± 0.0014
	Mactan	11	8	7	0.927 ± 0.067	0.0109 ± 0.0063	4	3	0.709 ± 0.099	0.0048 ± 0.0031
	Total	57	36	-	0.955 ± 0.017	0.0097 ± 0.0052	15	-	0.756 ± 0.039	0.0027 ± 0.0019

Sampling sites, number of individuals genotyped (No. gen.), number of haplotypes (No. hap.), number of unique haplotypes (No. uni.), gene diversity (*h* ± SD) and nucleotide diversity (*π* ± SD) statistics are listed.

**Table 5:** **Population pairwise*F***_**st**_**s of*Corythoichthys haematopterus*lineage A and B**

		Lineage A			Lineage B			
		Morode	Bounotsu	Kin	Kin	Bisezaki	Kuroshima	Mactan
Lineage A	Morode		- / -	- / -				
	Bounotsu	−0.001 / -0.0297		- / -				
	Kin	0.0392 / 0.0409	0.02286 / 0.04728					
Lineage B	Kin					- / -	- / -	+ / +
	Bisezaki				0.09218 / 0.02919		- / -	+ / +
	Kuroshima				−0.03686 / -0.03191	0.06622 / 0.01605		+ / +
	Mactan				0.35663 / 0.2297	0.16684 / 0.23522	0.33559 / 0.27719	

Population pairwise *F*_st_ estimates between sampling site, based on partial cytochrome b (*left*) and 16S rRNA (*right*) region of Cor*ythoichthys haematopterus*, analyzed in lineage A and B separately. + and – indicates significant and non-significant difference at P < 0.05, respectively.

*C. haematopterus* morphological characters are shown in Table 6. Comparing numerical characters of lineage A with those of lineage B, the range of most characters completely or partially overlapped with each other [pectoral fin rays: mode (range) = 14, 15 (14–15) and 16 (15–17), caudal fin rays: 10 (10) and 10 (10), trunk rings: 17 (17–18) and 17 (16–18); tail rings: 37, 38 (37–38) and 34 (31–37) in lineage A (n = 4) and B (n = 19), respectively], except for the number of dorsal fin rays where that of lineage A was less than that of lineage B [28 (28) and 30 (29–33) in lineage A and B, respectively].

**Table 6:** **Morphological characters of*Corythoichthys haematopterus***

Sampling site	Lineage	Standard length (mm)	Number of fin rays			Number of body rings	
			Pectoral fin	Dorsal fin	Caudal fin	Trunk	Tail
Morode	A	125.2	14	28	10	18	38
	A	131.7	15	28	10	17	37
	A	129.3	14	28	10	17	38
Kin	A	141.2	15	28	10	17	37
	B	124.1	16	29	10	17	35
	B	106.9	16	29	10	18	32
Bisezaki	B	125.2	16	31	10	18	32
	B	131.7	15	30	10	18	35
	B	129.3	17	33	10	17	37
Kuroshima	B	129.8	16	30	10	17	36
	B	83.7	17	32	10	17	37
	B	79.0	17	32	10	17	36
Mactan	B	111.0	16	29	10	17	34
	B	104.8	16	29	10	16	34
	B	109.9	16	31	10	17	36
	B	110.4	16	30	10	17	36
	B	122.4	15	30	10	17	31
	B	110.9	16	29	10	17	34
	B	103.8	17	30	10	17	35
	B	100.0	15	30	10	17	34
	B	103.0	16	29	10	16	34
	B	109.8	17	29	10	17	34
	B	114.7	15	29	10	17	35

## Discussion

The present study demonstrates that there are two distinct *Corythoichthys haematopterus* lineages inhabiting the Japanese archipelago and the Philippines. The distribution of lineage A was restricted to the main islands of Japan and a site in Okinawa Island. However, lineage B has a broad range from Okinawa Island southward, indicating that some biogeographic boundary exists between the two lineages. The genetic divergence between lineages (ca. 14% K2P + ***Γ*** distance for 16S rRNA region) is similar to or even higher than at the interspecific level: a phylogenetic tree based on 16S rRNA showed that *C. haematopterus* does not form a monophyletic group, two congeneric pipefishes, *C. flavofasciatus* and *C. schultzi*, were identified as sister groups to lineage B. Assuming a molecular clock for cytochrome *b* gene, calibrated at 1.4% sequence divergence per million years estimated from a study of seahorses sampled either side of the Isthmus of Panama (Casey et al. 2004), the genetic distance between the two lineages (ca. 23% TN93 + ***Γ*** + *I* distance for cytochrome *b* region) indicates that two lineages diverged in 16.6 Ma ago. Consequently, these results strongly suggest that the two *C. haematopterus* lineages should be treated as two species rather than as two divergent populations within a single species.

This view is well supported by the fact that the lineage B sequence data is very similar to that of the congeneric pipefish, *C. intestinalis*, whereas that of *C. haematopterus* is included in the lineage A clade. This indicates that the *C. haematopterus* lineage B should be a taxonomically recognized species, *C. intestinalis*, rather than either an unrecognized sub-species of *C. haematopterus* or an undescribed cryptic species. Therefore, the present study provides the first record of *C. intestinalis* in Japan where four species of *Corythoichthys* pipefishes have been recognized thus far (Nakabo 2002). The northern limit of *C. intestinalis* distribution, to date, was Northern Mariana Island (15°N) and south of Luzon Island (14°N) in the Pacific (Froese and Pauly 2013). Therefore the present study expands the distribution of *C. intestinalis* further north (about 1,200 km north) than previously recognized. Although it is not clear whether *C. intestinalis* inhabits southern Japan because of a recent range expansion or merely by the misidentification of *C. intestinalis* as *C. haematopterus*, our morphological assessment of the two lineages suggests that the latter is a plausible scenario.

Dawson (1977) who examined various morphological traits of 10 *Corythoichthys* pipefish species, mentioned that *C. haematopterus* and *C. intestinalis* are distinguishable based on the number of trunk rings (mode is 17 in the former and 16 in the later) or body marking differences. However, it has also been reported that the number of trunk rings varies among individuals of a single species (range is 16–18 and 15–17 in *C. haematopterus* and *C. intestinalis*; Dawson respectively; Dawson 1977), indicating that the number of trunk rings does not conclusively distinguish these two species. In addition, body coloration and markings exhibit intraspecific variation even in a single population (Dawson 1977; Kuiter 2003). The present study revealed that the number of dorsal fin rays differs between lineages (28 in lineage A and 29–33 in lineage B). However, Dawson (1977) reported considerable overlap in *C. haematopterus* and *C. intestinalis* (23–33 and 26–32, respectively). Consequently, such huge intraspecific variation in the characters employed for species recognition would lead to incorrect identification. Moreover, the genus *Corythoichthys*, which comprises at least 23 species, is distributed over a broad Indo-Pacific range includes members of species-complexes that were previously treated as a single widespread taxon (Kuiter 2003). Further morphological studies, in combination with molecular techniques, i.e. DNA barcoding, covering a diversity of species and broad geographical ranges are necessary to identify diagnostic characters for establishing a comprehensive taxonomy of the genus (Hebert et al. 2003). In general, the taxonomy and distribution of most syngnathid species remains poorly defined. Accurate identification of different species based on a reliable set of characteristics is necessary for planning effective management and conservation strategies.

The geographical distribution of the two lineages did not overlap each other, except for the Kin site where they cohabited on the vertical surface of a wharf (0–7 m depth). In their natural habitat, lineage A inhabited slopes consisting of boulder and bedrock around 2–10 m depth, whereas lineage B dwelled in seagrass meadows in shallow reef lagoons (less than 1 m depth). Clearly the two lineages differ in habitat preferences and are consequently spatially isolated from each other. However, artificial structures, such as wharves and breakwaters, at the Kin site provide both shallow and deep habitats within a small spatial scale, allowing the two lineages to cohabit and breed adjacently. There are ample examples that artificial habitat construction and alteration disrupts reproductive isolation among closely related species that are spatially isolated from each other in the natural environment (Arnold 1997). For instance, three species of greenlings (*Hexagrammos agrammus*, *H. octogrammus*, and *H. otakii*), which are reproductively isolated from each other in their natural habitat because of differences in depth and spawning substrate (e.g. seaweed or bryozoans) preferences, can hybridize in artificial environments consisting of steep slopes of complexly stacked concrete structures near a breakwater (Munehara et al. 2000; Kimura and Munehara 2010). Such an artificial environment would create a mosaic-habitat consisting of shallow and deep environments with diverse spawning substrate types, and consequently, increase sympatric greenling encounters. At present, however, it is not known if the two *C. haematopterus* lineages actually hybridize or if they are reproductively isolated from each other in the Kin site, because the present study only analyzed mitochondrial DNA markers. Further work should be carried out using nuclear DNA markers for elucidating the occurrence of hybridization and its fitness consequences for species conservation.

Two *C. haematopterus* lineages exhibited different population genetic structures. Low nucleotide and high gene diversity (as appeared in the star-shaped haplotype network of cytochrome *b)* in lineage A suggests that any previous phylogeographical structure in northwest Pacific populations has been erased by more recent range-expansion events (Avise 2000). During the Last Glacial Maximum (LGM; 16–21 ka), mean surface water temperature in the present Kuroshio Current region decreased to about 6°C, approximately 12°C colder than today (Oba and Murayama 2004). Considering the present distribution of *C. haematopterus*, between 35°N and 30°S in the Indo-Pacific (Froese and Pauly 2013), and their preference for warmer environments, it is highly probable that such a low temperature during the LGM lead to local extinctions and/or range retreats in *C. haematopterus* populations inhabiting the Japan Islands. Thereafter, recolonization and range expansion would have occurred around the Japan Islands when regional water temperatures drastically increased during the early Holocene (ca. 10 ka; Oba and Murayama 2004). In the tropical Pacific Ocean, sea surface temperatures probably did not change much during glacial cycles (CLIMAP Project Members 1976), although sea levels changed drastically by up to 120 m below present levels (Voris 2000). Large values of both gene and nucleotide diversity, together with a bush-like haplotype network in *C. haematopterus* lineage B can be attributed to a long evolutionary history in a large stable population (Grant and Bowen 1998).

The present study also showed that the levels of genetic variation among populations is low in both *C. haematopterus* lineages. Notably, a significant genetic divergence was confirmed between Mactan and the other lineage B populations. This genetic homogeneity in southern Japan likely reflects frequent gene flow among the populations and is attributable to the dispersal of pelagic larvae by the Kuroshio Current. However, previous studies that examined the population genetic structure of marine animals with a pelagic larval phase in the area of the Kuroshio Current have reported that two deep straits, the Tokara Gap and Kerama Gap (Figure 1) that divide the Japan Islands and the Ryukyu Islands into three parts, act as geographic barriers for larval dispersal resulting in genetic differentiation among populations (Ogoh and Ohmiya 2005; Kojima et al. 2006; Yorifuji et al. 2012). The difference in genetic population structure between *C. haematopterus* and those marine animals can be explained by differences in the length of the pelagic larval phase: the *C. haematopterus* pelagic larval phase is long (ca. 1 month) compared with those marine animals (a few days to a week), and enables them to disperse beyond these straits. This may indicate that not only the hydrodynamic characters of ocean currents but also the characters of pelagic larvae (e.g. larval duration) should be taken into account when determining larval dispersal and its effect on population genetic structure.

In conclusion, population genetic analysis of *C. haematopterus* in the northwest Pacific by mitochondrial DNA sequences indicates the existence of two genetically differentiated lineages, lineage A and B. We consider these two distinctive species of very similar appearance, *C. haematopterus* and *C. intestinalis*. The two lineages are speculated to have different biogeographic histories: rapid population expansion after a bottleneck event in lineage A and a long evolutionary history in a large stable population in lineage B, indicating that past climatic events and contemporary oceanographic features have played major roles in establishing the population genetic structure of *C. haematopterus*. Although the levels of genetic variation among populations was low in both lineages in the northwest Pacific, additional phylogeographic research with faster evolving genetic markers and intensive sampling covering the entire distributional range is required to elucidate the overall population genetic connectivity and inform conservation strategies for this species.

## Electronic supplementary material

Additional file 1: **Polymorphic nucleotide sites and haplotype frequency of partial mitochondrial DNA cytochrome*b*gene.** Polymorphic nucleotide sites and haplotype frequency of partial mitochondrial DNA cytochrome *b* gene (589 bp) detected in 108 individuals of *Corythoichthys haematopterus*. The number in parentheses indicates the number of fish collected in Sesoko. (PDF 26 KB)

Additional file 2: **Polymorphic nucleotide sites and haplotype frequency of partial mitochondrial 16S rRNA gene.** Polymorphic nucleotide sites and haplotype frequency of partial mitochondrial 16S rRNA gene (528 bp) detected in 108 individuals of *Corythoichthys haematopterus*. The number of parentheses indicates the number of fish collected in Sesoko. (PDF 24 KB)
