# Supplementary material for: Population genetic structure of the messmate pipefish Corythoichthys haematopterus in the northwest pacific: evidence for a cryptic species
Source: Springerplus. 2013 Aug 28;2:408. doi: 10.1186/2193-1801-2-408 (PMC3765599; doi:10.1186/2193-1801-2-408)
Supplement: Supplementary file 1 — Additional file 1: Polymorphic nucleotide sites and haplotype frequency of partial mitochondrial DNA cytochromebgene. Polymorphic nucleotide sites and haplotype frequency of partial mitochondrial DNA cytochrome b gene (589 bp) detected in 108 individuals of Corythoichthys haematopterus. The number in parentheses indicates the number of fish collected in Sesoko. (PDF 26 KB) [file 40064_2013_478_MOESM1_ESM.pdf]

Additional fiile 1: Polymorphic nucleotide sites and haplotype frequencies of partial mitochondrial DNA cytochrome b gene (589 bp) detected in 108 individuals of *Corythoichthys haematopterus*. The number in parentheses indicates the number of fish collected in Sesoko.

Haplo-type	Nucleotide position
	1122334455667777899111111111111111111111111222222222222222222222233333333333333333333333333
	06251739581713692170112333444566689999999000111223444556677899001111223334444555677788899
	3251069258403940134679258347038147395847958170349251470369458403929 47
1	TTCACACCCACCCTCGCTCCCCCCAGGCCCCGACCGCTGCGACTCCAATCTAAGCCCTTATGATATGACGTAGACAGACATCCCCCGC
2	··A·····················G·························
3	···············A························A·····················G·························
4	··A····················AG·························
5	··A·····················GC························
6	··A·····················G·········A···············
7	··A·····················G··············G··········
8	··A·····················G·························
9	··A·T···················G·························
10	··A·····················G·························
11	··························A·············A·····················G············G············
12	·················G······················A·····················G·························
13	··A·····················G·························
14	··A·····················G·························
15	··A·····················G·························
16	··A···
17	··A···
18	···G····································A·····················G·························
19	··A·····················G·························
20	···············A························A·····················G················G········
21	··A··········G··········G·························
22	··A·····················G··········T·····G········
23	··A·····················G·························
24	······················T·················A·····················G·························
25	ACT··CTTTTT·TATCTAGA·T····C·TGTTGTAATCTTATTAGTGGCGC··ATAACC·CCT·CCACTACGCGTTA·T··G··TTCA
26	ACT··CTTTTT·TATCTAGA·T····C·TG·TGTAATCTTATTAGTGGCGC·GATAACC·CCT·CCACTACGCGTTA·T··G··TTCA
27	ACT··CTTTTT·TATCTAGA·T····C·TG·TGTAATCTTATTAGTGGCGC··ATAACC·CCT·CCACTACGCGTTA·T··G··TTCA
28	ACT··CCTTTT·TATCTAGA·T····C·TG·TGTAATCTTATTAGTGGCGC··ATAACC·CCT·CCACTACGCGTTA·T··G··TTCA
29	ACT··CTTTTT·TATCTAGA·T····C·TG·TGTAATCTTATTAGTGGCGC··ATAACC·CCT·CCA·TACGCGTTA·T··G··TTCA
30	AC···CTTTTT·TATCTAGA·T·T··C·TG·TGTAATCT·ATTAGTGGCGC··ATAACC·CCT·CCACTACGCGTTA·T·CA··TTCA
31	ACT··CTTTTT·TATCTAGA·T····C·TG·TGTAATCTTATTAGTGGCGC··ATAACC·CCT·CCACTACGCGTTA·T··G··TTCA
32	AC···CTTTTT·TATCTAGA·T·T··C·TG·TGTAATCT·ATTAGTGGCGC··ATAACC·CCT·CCACTACGCGTTA·T·CG··TTCA
33	ACT·TCTTTTT·TATCTAGA·T····C·TG·TGTAATCTTATTAGTGGCGC··ATAACC·CCT·CCACTACGCGTTA·T··G··TTCA
34	ACT·TCTTTTT·TATCTAGA·T····C·TG·TGTAATCTTATTAGTGGCGC··ATAACC·CCT·CCACTACG·GTTA·T··G··TTCA
35	AC···CTTTTT·TATCTAGA·T·T··CTTG·TGTAATCT·ATTAGTGGCGC··ATAACC·CCT·CCACTACGCGTTA·T·CG··TTCA
36	AC···CTTTTT·TATCTAGA·T·T··C·TG·TGTAATCT·ATTAGTG·CGC··ATAACC·CCT·CCACTACGCGTTA·T·CG··TTCA
37	AC···CTTTTT·TATCTAGA·T·T··C·TG·TGTAATCT·ATTAGTGG·GC··ATAACC·CCT·CCACTACGCG·TA·T·CG··TTCA
38	AC···CTTTTT·TATCTAGA·T···AC·TG·TGTAATCT·ATTAGTCGCGC··ATAACC·CCT·CCACTAC·CGTTA·T·CG··TTCA
39	AC···CTTTTT·TATCTAGA·T···AC·TG·TGTAATCT·ATTAGTGGCGC··ATAACC·CCT·CCACTAC·CGTTA·T·CG··TTCA
40	AC···CTTTTT·TATCTAGA·T·T··C·TC·TGTAATCT·ATTAGTGGCGC··ATAACC·CCT·CCACTACGCGTTA·T·CG··TTCA
41	AC···CTTTTT·TATCTAGA·T·T··C·TG·TGTAATCT·ATTAGTGGCGC··ATAACC·CCT·CCATTACGCGTTA·T·CG··TTCA
42	ACT··CTTTTT·TATCTAGA·T····C·TG·TGTAATCTTATTAGTGGCGC··ATAACC·CCT·CCACTACGCGTTA·T··A··TTCA
43	AC···CTTTTT·TATCTAGA·T·T··C·TG·TGTAATCT·ATTAGTGGCGC··ATAACC·CCT·CCACTACGCG·TA·T·CG··TTCA
44	ACT··CTTTTTTTATCTAGA·T····C·TG·TGTAATCTTATTAGTGGCGC··ATAACC·CCT·CCACTACGCGTTA·T··G··TTCA
45	AC···CTTTTT·TATCTAGA·T·T··C·TG·TGTAATCT·ATTAGTGGCGC··ATAACC·CCT·CCACTACGCGTTA·T·CG··TTCA
46	ACT··CTTTTT·TATCTAGA·T····C·TG·TGTAATCTTATTAGTGGCGC··ATAACC·CCT·CCACTACGCGTTA·T··G··TTCA
47	ACT··CTTTTT·TATCTAGA·T····C·TG·TGTAATCTTATTAGTGGCGC··ATAACC·CCT·CCACTACGCGTTA·T··G··TTCA
48	AC···CTTTTT·TATCTAGA·T·TG·C·TG·TGTAATCT·ATTAGTGGCGC··ATAACC·CCT·CCACTACGCGTTA·T·CG··TTCA
49	AC···CTTTTT·TATCTAGA·T·T··C·TG·TGTAATCT·ATTAGTGGCGC··ATAACC·CCT·CCACTACGCGTTA·T·CG·TTTCA
50	AC···CTTTTT·TATCTAGA·T·T··C·TG·TGTAATCT·ATTAGTGGCGC··ATAACC·CCT·CCACTACGCGTTA·T·CGT·TTCA
51	ACT··CTTTTT·TATCTAGA·T····C·TG·TGTAATCTTATTAGTGGCGC··ATAACC·CCT·CCACTACGCGTTA·T··G··TTCA
52	AC···CTTTTT·TATCTAGA·T·T··C·TG·TGTAATCT·ATTAGTGGCGC··ATAACC·CCT·CCACTACGCG·TA·T·CG··TTCA
53	ACT CTTTTT TATCTAGATT C TG TGTAATCTTATTAGTGGCGC··ATAACC·CCT·CCACTACGCGTTA·T··G··TTCA
54	AC···CTTTTT·TATCTAGA·T···AC·TG·TGTAATCT·ATTAGTGGCGC··ATAACCGCCT·CCACTACGCGTTA·T·CG··T·CA
55	AC···CTTTTT·TATCTAGA·T·T··CTTG·TGTAATCT·ATTAGT·GCGC··ATAACC·CCT·CCACTACGCGTTA·T·CG··TTCA
56	AC···CTTTTT·TATCTAGA·T···AC·TG·TGTAA·CT·ATTAGTGGCGC··ATAACC·CCT·CCACTACGCGTTA·T·CG··T·CA
57	AC···CTTTTT·TATCTAGA·T·T··C·TG·TGTAATCT·ATTAGTGGCG···ATAACC·CCT·CCACTACGCGTTA·T·CG··TTCA
58	AC···CTTTTT·TATCTAGA·T·T··C·TG·TGTAATCT·ATTAGTGGCGC··GTAACC·CCT·CCACTACGCGTTA·T·CG··TTCA
59	AC···CTTTTT·TATCTAGA·T···AC·TG·TGTAATCT·ATTAGTGGCGC··ATAACC·CCT·CCACTACGCGTTA·T·CG··T·CA
60	AC···CTTTTT·TATCTAGA·T···AC·TA·TGTAATCT·ATTAGTGGCGC··ATAACCGCCT·CCACTACGCGTTA·T·CG··T·CA

Additional file 1: (*Continued*)

Haplo-type	Nucleotide position	Sampling site						Total	Frequency
	4 4 4 4 4 4 4 4 4 4 4 4 4 4 4 4 4 4 4 4 4 4 4 4 4 5 5 5 5 5 5 5 5 5 5 5 5 5 5 5 5 5 5 5 5 5 5 5<br>0 0 0 0 0 1 1 2 2 2 3 3 4 5 5 6 6 6 7 8 8 8 9 9 9 1 1 2 2 2 3 3 4 4 5 5 5 5 5 6 6 6 6 7 7 8 8 8<br>0 3 6 7 9 5 8 1 2 4 0 9 5 1 4 3 6 9 8 1 4 7 0 3 6 1 4 6 7 9 5 9 1 4 3 4 6 7 9 3 6 8 9 1 7 6 7 9	Morode	Bounotsu	Kin	Bisezaki	Kuroshima	Mactan		
1	TCAGTCGTAT ACGCATAGTCCTGTTCTCTATGAGTCAGACGTCAAGCC	2						2	0.019
2	· G · · ·	2						2	0.019
3	· ·	1						1	0.009
4	· ·	1						1	0.009
5	· ·	1						1	0.009
6	· ·	1						1	0.009
7	· A · · · · · · ·	1						1	0.009
8	· T ·	1						1	0.009
9	· ·	4	3					7	0.065
10	· ·	8	6	3				17	0.157
11	· ·		2					2	0.019
12	· ·		1					1	0.009
13	· A ·		1					1	0.009
14	· · · · · G ·		1					1	0.009
15	· C · · · · · · · · · · · · · · · · ·		1					1	0.009
16	· A · · · · · · · · · ·		1					1	0.009
17	· T ·		1					1	0.009
18	· ·		1	1				2	0.019
19	· · · · · · · · · · G ·		1	1				2	0.019
20	· ·			1				1	0.009
21	· ·			1				1	0.009
22	· ·			1				1	0.009
23	· G · · · · · · · · · · ·			1				1	0.009
24	· C · · · · · · · · · · · · ·			1				1	0.009
25	C · G · A · ACGC C ·AAGCGT · TAC · CC · CTGTC · · CCTG · GA · C · · CTA			1				1	0.009
26	C · G · A · ACGC C ·A · GCGT · TAC · CC · CTGTC · · CCTG · GA · C · G · CTA			1				1	0.009
27	C · G · A · A · GC C ·A · GCGT · TAC · CC · CTGTC · · CCTG · GA · C · G · CTA			1				1	0.009
28	C · G · A · ACGC C ·A · GCGT · TAC · CC · CTGTC · · CCTG · GA · C · · · CTA			1				1	0.009
29	C · G · A · ACGC C ·A · GCGT · TAC · CC · CTGTC · · CCTG · GA · C · G · CTA			1				1	0.009
30	C · G · A · ACGC C ·A · GCGT · TAC · CC · CTGTC · · CCT · · GA · C · G · CTA			1				1	0.009
31	C · G · A · ACGC C ·A · GCGT · TAC · CC · CTGTC · · CCTG · GA · C · · · CTA			2	2 (1)	5		9	0.083
32	C · G · A · ACGC C ·A · GCGT · TAC · CC · CTGTC · · CCT · · GA · C · · · CTA			1		1		2	0.019
33	C · G · A · ACGC C ·A · GCGT · TAC · CC · CTGTC · · CCTG · GA · C · G · CTA				1			1	0.009
34	C · G · A · ACGC CTA · GCGT · TAC · CC · CTGTC · · CCTG · GA · C · G · CTA				1			1	0.009
35	C · G · A · ACGC C ·A · GC · T · TA · · CC · CTGTC · · CCT · · GA · C · G · CTA				1			1	0.009
36	C · G · A · ACGC C ·A · GCGT · TAC · CC · CTGTC · · CCT · · GA · C · G · CTA				1			1	0.009
37	C · G · A · ACGC C ·A · GCCT · TAC · CC · CTGTC · · CCTG · GA · C · G · CTA				1			1	0.009
38	C · G · A · ACGC C ·A · · CGT · TAC · CC · CTGTCA · CCTG · GA · C · · · CTA				1			1	0.009
39	C · G · A · ACGC C ·A · · CGT · TAC · CC · CTGTCAGCCTG · GA · C · · · CTA				1			1	0.009
40	C · G · A · ACGC C ·A · GCGT · TAC · C · · CTGTC · · CCT · · GA · C · G · CTA				1			1	0.009
41	C · G · A · ACGC C ·A · GCGT · TAC · CC · CTGTC · · CCT · · GA · C · G · CTA				1 (1)			1	0.009
42	C · G · A · ACGC C ·A · GCGT · TAC · CC · CTGTC · · CCT · · GA · C · · · CTA				1 (1)			1	0.009
43	C · G · A · ACGC C ·A · GCGT · TAC · CC · CTGTC · · CCT · · GA · C · G · CTA				1 (1)			1	0.009
44	C G A ACGC G A GCGT TAC CC CTGTC CCTG GA C G CTA				1			1	0.009
45	C · G · A · ACGC C ·A · GCGT · TAC · CC · CTGTC · · CCT · · GA · C · G · CTA				4	1	3	8	0.074
46	C · G · A · ACGC C ·A · GCGT · TAC · CC · CTGTC · · CCTG · GA · C · G · CTA				1	2		3	0.028
47	C · G · A · ACGC C ·A · GCGT · TACACC · CTGTC · · CCTG · GA · C · · · CTA					3		3	0.028
48	C · G · A · ACGC C ·A · GCGT · TAC · CC · CTGTC · · CCT · · GA · C · G · CTA					1		1	0.009
49	C · G · A · ACGC C ·A · GCGT · TAC · CC · CTGTC · · CCT · · GA · C · G · CTA					1		1	0.009
50	C · G · A · ACGC C ·A · GCGT · TAC · CC · CTGTC · · CCT · · GA · C · G · CTA					1		1	0.009
51	C · G · A · ACGC C ·A · GCGT · TAC · CC · CTGTC · · CCTG · GA · CTG · CTA					1		1	0.009
52	C · G · A · ACGC C ·A · GCGT · TAC · CC · CTGTC · · CCTG · GA · C · G · CTA					1		1	0.009
53	C · G · A · ACGC C ·A · GCGT · TAC · CC · CTGTC · · CCTG · GA · C · G · CTA					1		1	0.009
54	C · G · A · ACGC C ·A · GCGT · TACACC · · TGTC · · CCTG · AA · C · G · CTA						2	2	0.019
55	C · G · A · ACGC C ·A · GCGT · TAC · C · · CTGTC · · CCT · · GA · C · G · CTA						1	1	0.009
56	C · G · A · ACGC C ·A · GCGT · TACACC · · TGTC · · CCTG · AA · C · · · CTA						1	1	0.009
57	C · GAA · ACGC C ·A · GCGT · TAC · CC · CTGTC · · CCT · · GA · C · G · CTA						1	1	0.009
58	C · G · A · ACGC C ·A · GCGT · TAC · CC · CTGTC · · CCT · · GA · C · G · CTA						1	1	0.009
59	C · G · A · ACGC C ·A · GCGTCTACACC · · TGTC · · TCTG · AA · C · G · CTA						1	1	0.009
60	C · G · A · ACGC C ·A · GCGT · TACACC · · TGTC · · CCTG · AA · C · G · CTA						1	1	0.009
