# Supplementary material for: Population genetic structure of the messmate pipefish Corythoichthys haematopterus in the northwest pacific: evidence for a cryptic species
Source: Springerplus. 2013 Aug 28;2:408. doi: 10.1186/2193-1801-2-408 (PMC3765599; doi:10.1186/2193-1801-2-408)
Supplement: Supplementary file 2 — Additional file 2: Polymorphic nucleotide sites and haplotype frequency of partial mitochondrial 16S rRNA gene. Polymorphic nucleotide sites and haplotype frequency of partial mitochondrial 16S rRNA gene (528 bp) detected in 108 individuals of Corythoichthys haematopterus. The number of parentheses indicates the number of fish collected in Sesoko. (PDF 24 KB) [file 40064_2013_478_MOESM2_ESM.pdf]

Additional file 2: Polymorphic nucleotide sites and haplotype frequency of partial mitochondrial 16S rRNA gene (528 bp) detected in 108 individuals of *Corythoichthys haematopterus*. The number in parentheses indicates the number of fish collected in Sesoko.

Haplotype	Nucleotide position	Sampling site						Total	Frequency
	**367911111111111111111111122222222222222222222222222222222223333333333333444445<br>34200002244455888999990000000011112222223333344458889999001123455667822461<br>26782536904567027891234567801240235671458924810233479040575067786547286**	Morode	Bounotsu	Kin	Bisezaki	Kuroshima	Mactan		
1	CACGCTCGTCGACCAAGCGGACTTAATAAGTTDGCAAADCATTTAACCTGGAAGTGACDAATCAAGAACTCTTCG	1						1	0.009
2	···A····G··············	1						1	0.009
3	···A·········A·········	1						1	0.009
4	··G··A···················	1	1					2	0.019
5	·································A·····················A···················	1	1					2	0.019
6	···G·········A···················	1	1					2	0.019
7	···A···A···················	1	2					3	0.028
8	···A···················	15	13	5				33	0.306
9	··G··········A···················		1					1	0.009
10	·······································G···············A···················			1				1	0.009
11	···A·A·················			1				1	0.009
12	···A··········G········			1				1	0.009
13	·G···A···················			2				2	0.019
14	T·AATCTAAT··AA·TA AAGTGCCC·GTTCCCA·GGGT·CCCCTTTTGACG·CAATGCG·CTGC··TTGTA·TA			1				1	0.009
15	T·AATCTAAT··AA·TATAAGTGCCC·GT CCCA·GGGT·CCCCTTTTGACG·CAATGCG·CTGC··TTGTA·TA			1				1	0.009
16	T·AATCTAAT··AA·TATAAGTGCCC·GTTCCCA·GGGT·CCCCTTTTGACG·CAACGCG·CTGC··TTGTA·TA			1				1	0.009
17	T·AATCTAAT··AA·TATAAGTGCCC·GTTCCCA·GGGT·CCCCTTTTGACG·CAATGCG·CTGC··TTGTA·TA			4	5 (1)	11		20	0.185
18	T·AATCTAAT··AA·TATAAGTGCCC·GTCCCCA·GGGT·CCCCTTTTGACG·CAATGCG·CTGC··TTGTA·TA			2	10 (2)	4	4	20	0.185
19	T·AATCTAAT··AA·TATAAGTGCCCA·TTCCCA·GGGT·CCCCTTTTGACG·CAATGCG·CTGC··TTGTA·TA				1			1	0.009
20	T·AATCTAAT··AA·TATAAGTGCCC·GTTCCCATGGGT·CCCCTTTTGACG·CAATGCG·CTGC··TTGTA·TA				1 (1)			1	0.009
21	T·AATCTAAT··AA·TATAAGTGCCC··TTCCCA·GGGT·CCCCTTTTGACG·CAATGCG·CTGC··TTGTA·TA				1			1	0.009
22	T·AATCTAAT··AA·TATAAGTGCCC·GTTCCCA·GGGT·CCCCTTTTGAC··CAATGCG·CTGC··TTGTA·TA				1			1	0.009
23	T·AATCTAAT··AAGTATAAGTGCCC·GTCCCCA·GGGT·CCCCTTTTGACG·CAATGCG·CTGC··TTGTA·TA					1		1	0.009
24	T·AATCTAAT··AA·TATAAGTGCCC·GTCCCCA·GGGT·CCCCTTTTGACG·CAATGCG·CTGCA·TTGCA·TA					1		1	0.009
25	T·AATCTAAT··AA·TATAAGTGCCC·GTTCCCA·GGGT·CCCCTTTTGACG·CAATGCG·CTGC··TTGTACTA					1		1	0.009
26	T·AATCTAAT·GAA·TATAAGTGCCC·GTTCCCA·GGGC·CCCCTTTTGAC··CAATGCG·CTGC··TTGTA·TA						5	5	0.046
27	T·AATCTAATA·AA·TATAAGTGCCC·GTCCCCA·GGGT·CCCCTTTTGACG·CAATGCG·CTGC··TTGTA·TA						1	1	0.009
28	T·AATCTAAT··AA·TATAAGTGCCC·GTCCCCATGGGT·CCCCTTTTGACG·CAATGCG·CTGC··TTGTA·TA						1	1	0.009
